# INTERSECTIONS OF SEXUAL ORIENTATION AND GENDER IN SPOUSAL CAREGIVING: A FOCUS ON COGNITIVE LABOR

**DOI:** 10.1093/geroni/igac059.2546

**Published:** 2022-12-20

**Authors:** Sadie Giles, Toni Calasanti, Jing Geng, Brian de Vries

**Affiliations:** Virginia Tech, Blacksburg, Virginia, United States; Virginia Tech, Blacksburg, Virginia, United States; Virginia Tech, BLACKSBURG, Virginia, United States; San Francisco State University, Palm Springs, California, United States

## Abstract

Our presentation adopts a lens of intersecting inequalities based on gender and sexual orientation in exploring caregiving for a spouse or partner with dementia. How the division of labor shapes caregiving approaches is sometimes examined, but how this might differ by sexual orientation has not been explored. Previous research has often examined physical tasks and, to a lesser degree, emotion work. Here, we focus on cognitive labor (Daminger, 2019): the mental labor involved in managing the household and social relationships, including anticipating needs, identifying ways to address these, making decisions, and monitoring outcomes. This theoretical backdrop informs our analysis of data garnered from in-depth interviews conducted among gay/lesbian and heterosexual older adults (N=57) who care for their spouses with dementia. However couples may have divided these cognitive steps previously, caregivers must perform them all alone, given care receivers’ cognitive losses. Our analyses reveal that caregivers find being responsible for everything exhausting, but this manifests differently based on gender and sexual orientation. Heterosexual women, for instance, found making all the decisions problematic, whereas heterosexual men reported difficulty in anticipating needs. For the most part, the challenging issues for gay and lesbian caregivers were more varied, as their previous division of labor had been negotiated differently, without a reliance on traditional gender roles. Finally, we uncover a novel domain for cognitive labor: being the “memory keeper.” Predominantly undertaken by women, this work involved maintaining important memories and markers of the care receiver’s (and couple’s) past.

